# Clinical outcomes of tooth-supported monolithic zirconia vs. porcelain-veneered zirconia fixed dental prosthesis, with an additional focus on the cement type: a systematic review and meta-analysis

**DOI:** 10.1007/s00784-023-05219-4

**Published:** 2023-08-26

**Authors:** Shahed Shihabi, Bruno Ramos Chrcanovic

**Affiliations:** 1 Faculty of Odontology, Malmö University, Malmö, Sweden; 2 Department of Prosthodontics, Faculty of Odontology, Malmö University, Carl Gustafs Väg 34, 214 21 Malmö, Sweden

**Keywords:** Dental prosthesis, Tooth-supported prosthesis, Monolithic zirconia, Porcelain-veneered zirconia, Cement type, Cementation, Failure, Complications, Systematic review

## Abstract

**Purpose:**

To compare the failure rates and the prevalence of technical complications between full-coverage tooth-supported monolithic zirconia (MZ) and porcelain-veneered zirconia (PVZ) fixed dental prosthesis, based on a systematic literature review.

**Methods:**

An electronic search was performed in three databases, supplemented by hand searching. Several statistical methods were used.

**Results:**

Seventy-four publications reported 6370 restorations (4264 PVZ; 2106 MZ; 8200 abutment teeth; 3549 patients), followed up until 152 months. A total of 216 prostheses failed, and survival was statistically significant different between groups. PVZ had higher occurrence of complications than MZ; the difference was especially greater for either minor or major chipping. The difference in prevalence of either minor or major chipping was statistically significant for PVZ prostheses between cementation with glass ionomer and adhesive resin cement (higher), adhesive resin and resin-modified glass ionomer cement (RMGIC, higher), and between RMGIC (higher) and glass ionomer cement. For MZ the difference was significant only for minor chipping between RMGIC (higher) and adhesive resin cement. Abutment teeth to PVZ prostheses more often lost vitality. Decementation was not observed with RMGIC. Air abrasion did not seem to clinically decrease the decementation risk. The 5-year difference in the occurrence of minor or major chipping between MZ and PVZ prostheses was statistically significant, but nor for catastrophic fracture.

**Conclusion:**

Tooth-supported PVZ prostheses present higher failure and complication rates than MZ prosthesis. The difference in complications is striking when it comes to chipping.

**Clinical relevance:**

Awareness of the outcome differences between different types of zirconia prostheses is important for clinical practice.

**Supplementary Information:**

The online version contains supplementary material available at 10.1007/s00784-023-05219-4.

## Introduction

The development of yttria-stabilized tetragonal zirconia polycrystalline (Y-TZP) for dental purposes has resulted in a range of different products. The main advantages of Y-TZP include exceptional mechanical properties, biocompatibility, and resistance to corrosion [1, 2]. One of the greatest disadvantages of the Y-TZP, at least when it comes to dental restorations, is the less than desirable translucency of the material. Translucency is important, as a certain degree of translucency of the dental ceramic is needed to let the natural background color shine throughout the translucent material, so the dental restoration can present a more natural appearance [3].

There are today two main categories of dental prostheses made of Y-TZP: monolithic zirconia (MZ), in which the bulk of the restorations is made of zirconia and external stains are painted in order to better copy the natural colors of teeth, and porcelain-veneered zirconia (PVZ), in which a framework of zirconia is fabricated, upon which veneering porcelain is applied [4]. Prostheses made of MZ are stronger, but present a compromised aesthetic aspect, while PVZ prostheses, with a core of Y-TZP and an outer layer of ceramic (also called as bilayer structure), better resemble natural teeth, although are vulnerable to chipping and delamination [2]. This may predispose PVZ to a higher prevalence of clinical technical complications.

Questions have been raised concerning the effect of cement type on the clinical outcomes, although a recent review failed to find evidence of difference in the complication patterns between adhesive and conventional cementation for zirconia and lithium disilicate tooth-supported crowns [5].

Therefore, the purpose of the present systematic review of the literature was to test the null hypothesis of no difference in the failure rates and the prevalence of technical complications between full-coverage tooth-supported MZ and PVZ fixed dental prosthesis (FDP). The review presented an additional focus on the effect of the type of cement used on the occurrence of complications.

## Materials and methods

This study followed the PRISMA Statement guidelines [6]. Registration in PROSPERO was undertaken (registration number CRD42022342097).

### Objective

The focused question was elaborated according to the PICO format (participants, interventions, comparisons, outcomes): In patients being rehabilitated with dental prosthetic restorations, is there a difference in the failure rate and in the prevalence of technical complications between monolithic zirconia and porcelain-veneered zirconia prostheses?

### Search strategies

An electronic search without time restrictions was undertaken in October 2021, with a complementary updated search in June 2023, in the following databases: PubMed/Medline, Web of Science, and Science Direct. The following terms were used in the search strategies: (tooth OR teeth OR tooth-supported) AND (dental prosthesis OR dental restoration) AND (monolithic zirconia OR porcelain-veneered zirconia OR porcelain fused to zirconia OR pressed on zirconia ceramics).

Due to the initial large number of search entries in Science Direct, the options “research articles” and “review articles” were selected in the filter “Article type.”

A manual search of all related prosthodontic and specialist dental and oral journals was performed. The reference list of the identified studies and the relevant reviews on the subject were also checked for possible additional studies. Gray literature was not searched.

### Inclusion and exclusion criteria

Eligibility criteria included clinical human studies, either randomized or not, providing information on the clinical outcomes of full-coverage tooth-supported MZ and/or PVZ FDPs. The minimum of follow-up was set to 6 months. Only publications written in English were considered for inclusion.

Combined tooth-implant-supported FDP cases were excluded, as well as zirconia copings to be used with removable partial dentures. Inlay-retained FDP cases were excluded, as they do not present the same quality of support as a conventional FDP. Cases of zirconia restorations aimed to be cemented on abutment teeth for partial removable dental prosthesis were excluded, as these are subjected to additional forces on the occlusal rest seat and from retentive clasps. Cases of partial crowns and endocrowns were excluded. Additional exclusion criteria were case reports, technical reports, animal studies, in vitro studies, and reviews papers.

### Study selection

Two reviewers independently screened the titles and abstracts of the entries (publications) resulted from the searches conducted in the three databases. The full text of a publication was obtained when this appeared to meet the inclusion criteria, or for when there were insufficient data in the title and abstract to make a clear decision, which was carried out independently by two reviewers. Any disagreements between the reviewers were solved by discussion.

The detection of duplicate references from different electronic databases was performed by using the RefWorks Reference Management Software (Ex Libris, Jerusalem, Israel).

### Quality assessment

The Quality Assessment Tool for Case Series Studies of the National Institutes of Health [7] was used for the quality assessment of the included studies. The studies were classified as “good” (at least 7 points—the least risk of bias; results are considered to be valid), “fair” (susceptible to some bias deemed not sufficient to invalidate its results), or “poor” quality (significant risk of bias).

### Definitions

Yttria-stabilized zirconia is a ceramic material, a white crystalline oxide of zirconium (ZrO_2_), with its crystal structure stabilized by the addition of yttrium oxide (Y_2_O_3_) or yttria [8].

Monolithic zirconia restorations were defined as those with the same chemical and physical properties throughout its thickness [9]. These are dental prostheses with a bulk of zirconia fabricated by computer-aided design and computer-aided manufacturing (CAD/CAM) and painted with external stains [4].

Porcelain-veneered zirconia restorations were defined as those with a zirconia framework enhanced with veneering porcelain [9].

Success was defined as a prothesis that had remained unchanged (no complication or intervention) over the observation period. Survival was defined as the cases in which the prothesis remained in situ, with the occurrence of any complication, while still in function. Prostheses removed or replaced were considered failed prostheses [10].

Biological complications included caries, loss of tooth vitality, periapical infection, mobility, and abutment loss. Technical complications included tooth fracture, loss of retention, framework fracture, and minor and major veneer chipping.

Chipping was defined as loss of ceramic substance, being classified as minor (managed chair-side, such as in-mouth polishing of the fracture) or major (usually sent to the dental lab for reparation or replaced by a new prosthesis) [11, 12].

Catastrophic fracture was defined as a fracture extending through the entire bulk of the restoration, namely, from the external to the inner surface.

“Unacceptable color” and “unacceptable anatomical form” of the restorations were considered neither biological nor technical complications, and therefore not considered for the present review.

### Data extraction

From the studies included in the final analysis, the following data were extracted (when available): year of publication, study design and setting, number of patients, patients’ age, type of material (MZ, PVZ), number of failed and placed prosthesis, jaws receiving the prosthesis (maxilla and/or mandible), type of prosthetic rehabilitation, opposing dentition, presence of smokers or bruxers, and follow-up time. Authors of the included studies were contacted for additional information in case of need for additional data.

### Analyses

Descriptive statistics were expressed in means, standard deviations (SD), and percentages; Kolmogorov–Smirnov test was used to evaluate normal distribution and Levene’s test to evaluate homoscedasticity. The comparison of continuous variables between two independent groups was done with Student’s t-test or Mann–Whitney, and Pearson’s chi-squared or Fisher’s exact test for the comparison of categorical variables. Comparison of prostheses survival between different groups was done with the log-rank (Mantel-Cox) test. Information for the period of prosthesis failure extracted from the included studies was used to calculate interval survival rate (ISR), and the cumulative survival rate (CSR) was calculated over the maximal period of follow-up reported, in a life-table survival analysis.

The estimated 5-year occurrence proportions of minor and major chipping, as well as of catastrophic fracture were calculated, by assuming constant event rates. The total exposure time of the prostheses of the studies was calculated, and from this the estimated annual rate per 100 prosthesis years and the estimated occurrence after 5 years for each of these 3 outcomes were calculated. The 95% confidence intervals for the survival proportions were calculated using the 95% confidence limits of the event rates. A meta-analysis for each group was conducted using proportions with inverse-variance weights. The value of 0.5 were added to zero frequencies. Groups were then compared using a z-test.

The degree of statistical significance was considered *p* < 0.05. Data were statistically analyzed using the SPSS version 28 software (SPSS Inc., Chicago, IL, USA).

## Results

### Literature search

**Fig. 1:**
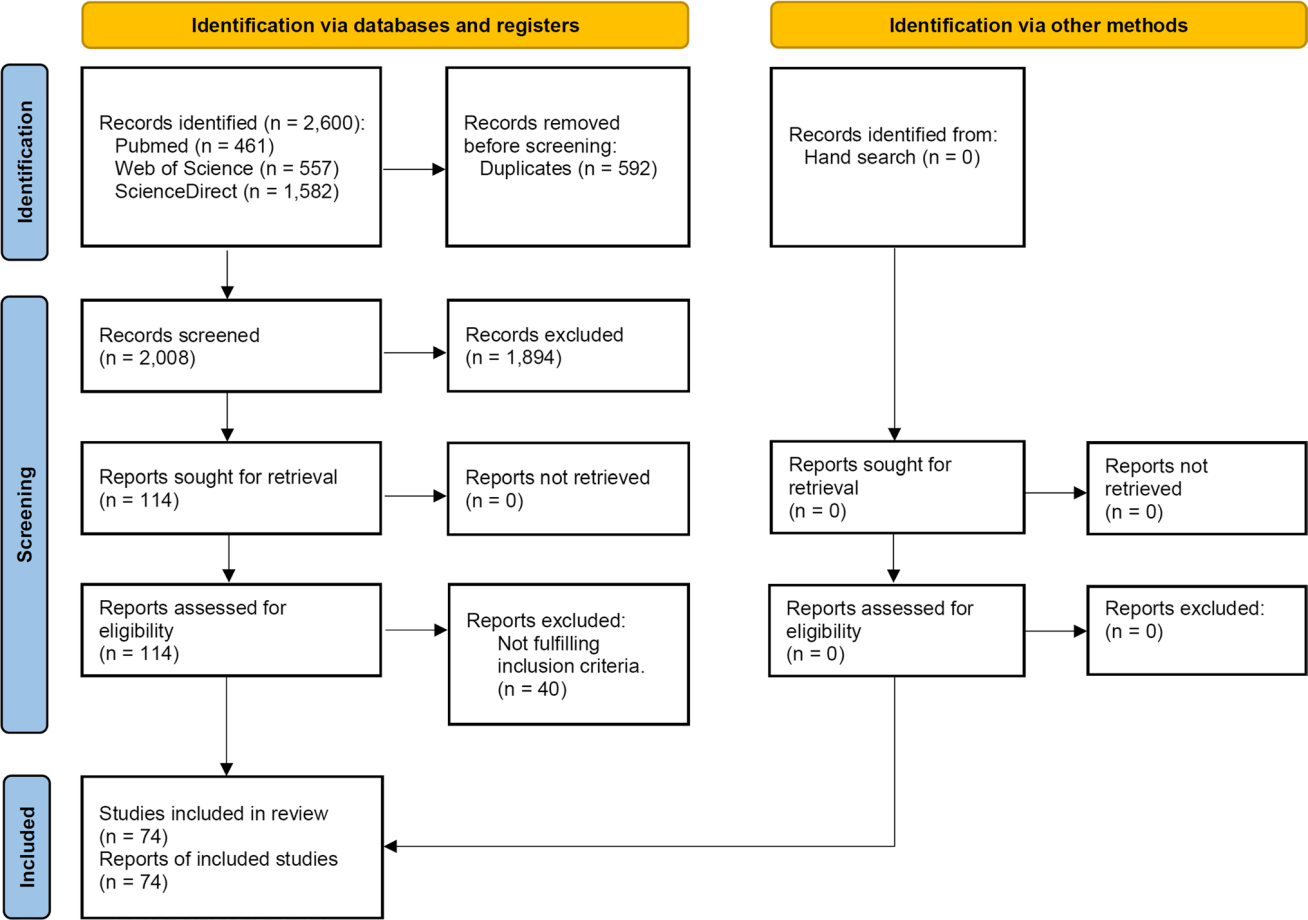
Study screening process

The study selection process is summarized in Fig. 1. The search strategy initially resulted in 2600 papers (461 in PubMed, 557 in Web of Science, and 1582 in Science Direct). A number of 592 articles were cited in more than one research of terms (duplicates). Two reviewers independently screened the abstracts for those articles related to the focus question. Of the resulted 2008 studies, 1894 were excluded for not being related to the topic. Hand searching of selected journals did not yield additional papers. The full-text reports of the remaining 114 articles led to the exclusion of 40 articles because they did not meet the inclusion criteria: shorter follow-up report with an already published longer follow-up report with the same cohort group of patients (*n* = 16), not enough or no clinical outcome data available (*n* = 4), no separate information on clinical outcomes between tooth- and implant-supported prostheses (*n* = 3), endocrowns (*n* = 3), inlay-retained restorations (*n* = 3), more than one type of restoration material included in the study, but no separate data on the zirconia restorations (*n* = 1), restorations made of zirconia-reinforced lithium silicate (*n* = 1), technical complications not investigated (*n* = 1), restorations in abutment teeth for partial removable dentures (*n* = 1), 3D-printed zirconia crowns (*n* = 1), no follow-up (*n* = 1), not providing clinical information regarding the number of prosthesis, but the number of prosthetic units (*n* = 1), lab study (*n* = 1), single-retainer prostheses (*n* = 1), partial crowns (*n* = 1), and study of post-cementation occlusion with no follow-up (*n* = 1). Thus, 74 publications were included in the review [13–86].

### Description of the studies

The 74 included clinical studies reported 6264 tooth-supported zirconia FDPs, supported by 8200 abutment teeth (pontics were not taken into consideration in this amount). The 3549 patients consisted of 1377 (43.1%) men and 1818 (56.9%) women, with no available information on gender for 354 patients.

Table 1 presents the summarized data of the included studies, separately between the two groups. The mean follow-up time of the group of PVZ was higher than for the group of MZ prostheses (*p* < 0.001; Mann–Whitney test). Women were more often than men rehabilitated with PVZ FDPs, the opposite happening with MZ FDPs (*p* < 0.001; Pearson’s chi-squared test). Most of the prostheses were single crowns, for both groups (67.7% of PVZ and 94.4% of MZ). Adhesive resin was most commonly used for cementation of the prostheses, and natural teeth were most often observed in the opposing arch occluding to the zirconia prostheses.

**Table 1:** Summarized data of the included studies—patients rehabilitated with tooth-supported porcelain-veneered zirconia and monolithic zirconia prostheses (information was not always available for all prostheses for all variables)

Variable	Veneered zirconia	Monolithic zirconia
Patients (***n***)/prostheses (***n***)/abutment teeth (***n***)	2410/4264/5939	1139/2106/2261
Age (years), mean ± SD (min–max)	50.4** ± **8.1 (16–89)	53.9 ± 9.5 (20–88)
Follow-up (months), mean ± SD (min–max)	48.2 ± 30.0 (6–152)	38.4** ± **19.7 (6–96)
Sex, ***n*** (%)		
Men/women	868 (40.2)/1290 (59.8)	509 (49.1)/528 (50.9)
Not available	252	102
Prosthesis, ***n*** (%)		
Men	1368 (36.7)	1085 (54.4)
Women	2363 (63.3)	911 (45.6)
Not available	643	
Abutment teeth per patient (***n***), mean ± SD (min–max)	2.4 ± 1.5 (1–19)	2.0 ± 2.7 (1–28)
Prosthesis type, ***n*** (%)		
Single crown	2,888 (67.7)	1,989 (94.4)
2-unit	24 (0.6)	21 (1.0)
3-unit	1,004 (23.5)	70 (3.3)
4-unit	188 (4.4)	15 (0.7)
5-unit	86 (2.0)	5 (0.2)
6-unit	46 (1.1)	1 (0.1)
7-unit	18 (0.4)	4 (0.2)
8-unit	5 (0.1)	1 (0.1)
10-unit	3 (0.1)	-
12-unit	2 (0.1)	-
Cement type, ***n*** (%)		
Glass ionomer	948 (23.7)	193 (9.2)
Resin-modified glass ionomer	410 (10.3)	343 (16.3)
Adhesive resin	2348 (58.8)	1555 (73.8)
Provisionally cemented	28 (0.7)	15 (0.7)
Zinc phosphate	259 (6.5)	-
Not available	271	-
Pre-cementation air-abrasion, ***n***/total (%)	910/4264 (21.3)	640/2106 (30.4)
Situation in the opposite arch, ***n*** (%)		
Natural teeth	1208 (59.8)	302 (96.8)
Amalgam/composite	188 (9.3)	6 (1.9)
Fixed partial denture	623 (30.8)	4 (1.3)
Removable partial denture	2 (0.1)	-
Not available	2243	1794
Prosthesis failure (***n***), failure/total (%)		
Prosthesis level	177/4264 (4.2)	39/2106 (1.9)
Patient level	106/2410 (4.4)	38/1139 (3.3)
Time of failure (months), mean ± SD (min–max)	38.6 ± 24.0 (1–132)	40.3 ± 27.4 (6–96)
Reasons for prosthesis failure, ***n*** (%)		
Abutment tooth loss (fracture or periodontal reasons)	40 (22.7)	18 (46.1)
Framework fracture	30 (17.0)	5 (12.8)
Fracture of connector	4 (2.3)	-
Secondary caries ^a^	22 (12.5)	4 (10.3)
Major chipping ^a^	58 (33.0)	2 (5.1)
Endodontic treatment ^a^	5 (2.8)	2 (5.1)
Decementation/retention loss ^a^	13 (7.4)	2 (5.1)
Core fracture	-	1 (2.5)
Persistent pain	1 (0.6)	-
Periapical fistula/granuloma	3 (1.7)	2 (5.1)
New treatment approach	-	3 (7.7)
Not available	1	-
Abutment tooth failure (***n***), failure/total (%)	45/5939 (0.8)	20/2261 (0.9)
Reasons for abutment failure, ***n*** (%)		
Fracture	25 (56.8)	16 (80.0)
Periodontal	12 (27.3)	2 (10.0)
Endodontic complication	2 (4.5)	2 (10.0)
Caries	5 (11.4)	-
Not available	1	-
Complication ^b^, event (% total prostheses)		
Tooth fracture	26 (0.6)	16 (0.8)
Vitality loss	61 (1.5)	6 (0.3)
Secondary caries	43 (1.0)	9 (0.4)
Periapical infection	7 (0.2)	1 (0.1)
Chip-off		
Minor	303 (7.1)	7 (0.3)
Major ^c^	110 (2.6)	2 (0.1)
Framework fracture	33 (0.8)	5 (0.2)
Decementation	57 (1.4)	21 (1.0)
Frequency by cement, ***n*** (%)		
Glass ionomer	15 (1.6)	1 (0.5)
Resin-modified glass ionomer	0 (0)	0 (0)
Adhesive resin	29 (1.3)	20 (1.3)
Provisionally cemented	0 (0)	0 (0)
Zinc phosphate	13 (5.0)	-

^a^For the cases that led to the replacement of the prosthesis, which was not always the case for this complication

^b^For the cases with the information available

^c^Not every major chipping led to prosthesis failure/replacement

In general, PVZ FDPs had a higher occurrence of complications than MZ FDPs. The difference was, in particular, greater when either minor or major chip-offs were considered. Decementation was more commonly seen in prosthesis cemented with adhesive resin than with glass ionomer, with no occurrences with resin-modified glass ionomer.

Table 2 shows the prevalence of minor and major chipping among tooth-supported PVZ and MZ prostheses, for the different types of cementations applied. If the factor time is not considered, the difference of the prevalence of chipping was statistically significant for PVZ prostheses between cementation with glass ionomer and adhesive resin cement (for minor chipping), resin-modified glass ionomer and adhesive resin cement (for both minor and major chipping), and between resin-modified glass ionomer and glass ionomer cement (for both minor and major chipping). For MZ the difference was significant only for minor chipping between resin-modified glass ionomer and adhesive resin cement.

**Table 2:** Prevalence of minor and major chipping among tooth-supported porcelain-veneered zirconia and monolithic zirconia prostheses, for the different types of cementations applied. Here are considered the cases with available information for both the cement type used and the occurrence of chipping. The unit is the prosthesis

Cement type	Veneered zirconia			Monolithic zirconia		
	Minor chipping	Major chipping	Total number prostheses	Minor chipping	Major chipping	Total number prostheses
	*n* (%)	*n* (%)	*n*	*n* (%)	*n* (%)	*n*
Glass ionomer	28 (3.0)	12 (1.3)	948	0 (0)	0 (0)	193
Resin-modified glass ionomer	80 (19.5)	33 (8.0)	410	6 (1.7)	0 (0)	343
Adhesive resin	162 (6.9)	53 (2.3)	2348	1 (0.1)	1 (0.1)	1,555
Provisionally cemented	0 (0)	0 (0)	28	0 (0)	1 (6.7)	15
Zinc phosphate	28 (10.8)	4 (1.5)	259	-	-	-
Total	298 (7.5)	102 (2.6)	3993	7 (0.3)	2 (0.1)	2,106
Difference in prevalence		***p* value**			***p* value**	
Glass ionomer × adhesive resin						
Minor chipping		< 0.001 ^a^			0.999^b^	
Major chipping		0.064^a^			0.999^b^	
Resin-modified glass ionomer × adhesive resin						
Minor chipping		< 0.001 ^a^			< 0.001 ^b^	
Major chipping		< 0.001 ^a^			0.999 ^b^	
Resin-modified glass ionomer × glass ionomer						
Minor chipping		< 0.001 ^a^			0.092^b^	
Major chipping		< 0.001 ^a^			^c^	

^a^Pearson’s chi-squared test

^b^Fishers’ exact test

^c^No events in either group

Table 3 shows the prevalence of decementation among tooth-supported PVZ or MZ prostheses, for the different types of cementations applied, between prostheses that had their inner surface air abraded or not. No cases of decementation were observed for prostheses cemented with resin-modified glass ionomer, regardless of whether air abraded or not. Air-abraded PVZ FDPs decemented more often when cemented with conventional glass ionomer cement.

**Table 3:** Prevalence of decementation among tooth-supported porcelain-veneered zirconia or monolithic zirconia prostheses, between prostheses with inner surface air abraded or not previously to cementation, for the different types of cementations applied. Here are considered the cases with available information for both the cement type used and the occurrence of decementation. The unit is the prosthesis

Cement type	Veneered zirconia			Monolithic zirconia		
Decementation, *n*/total (%)						
	No air-abrasion	Air-abrasion	*p* value	No air-abrasion	Air-abrasion	*p* value
Glass ionomer	8/770 (1.0)	7/178 (3.9)	0.012 ^b^	1/120 (0.8)	0/73 (0)	1.000 ^b^
Resin-modified glass ionomer	0/390 (0)	0/20 (0)	^d^	0/343 (0)	-	-
Adhesive resin	20/1,649 (1.2)	9/613 (1.5)	0.631 ^a^	12/1,003 (1.2)	8/552 (1.4)	0.672 ^a^
Provisionally cemented	0/28 (0)	-	-	-	0/15 (0)	-
Zinc phosphate	2/160 (1.3)	11/99 (11.1)	< 0.001 ^b^	-	-	-
Total	30/2997 (1.0)	27/910 (3.0)	< 0.001 ^a^	13/1466 (0.9)	8/640 (1.3)	0.440 ^a^
Difference in prevalence of decementation			***p* value**			***p* value**
Glass ionomer × adhesive resin						
No air abrasion			0.710 ^a^			1.000 ^b^
Air abrasion			0.063 ^b^			0.606 ^b^
Resin-modified glass ionomer × adhesive resin						
No air abrasion			0.021 ^b^			0.044 ^b^
Air abrasion			1.000 ^b^			^c^
Resin-modified glass ionomer × glass ionomer						
No air abrasion			0.057 ^b^			0.259 ^b^
Air abrasion			1.000 ^b^			^c^

^a^ Pearson’s chi-squared test

^b^ Fishers’ exact test

^c^ No cases cemented by resin-modified glass ionomer and air abraded

^d^ No events in both groups

Table 4 shows the prevalence of vitality loss among teeth supporting PVZ or MZ prostheses for the different types of cementations applied. In general, abutment teeth to PVZ prostheses more often lost vitality.

**Table 4:** Prevalence of vitality loss among teeth supporting porcelain-veneered zirconia or monolithic zirconia prostheses, for the different types of cementations applied. Here are considered the cases with available information for both the cement type used and the occurrence of vitality loss. The unit is the prosthesis

Cement type	Vitality loss, *n*/total (%)		*p* value ^a^
	Veneered zirconia	Monolithic zirconia	
Glass ionomer	16/948 (1.7)	2/193 (1.0)	0.753 ^c^
Resin-modified glass ionomer	1/410 (0.2)	0/343 (0)	1.000 ^c^
Adhesive resin	31/2262 (1.4)	4/1555 (0.3)	< 0.001 ^b^
Provisionally cemented	0/28 (0)	0/15 (0)	^d^
Zinc phosphate	8/259 (3.1)	-	-
Total	56/3907 (1.4)	6/2106 (0.3)	< 0.001 ^b^

^a^ Comparison in the prevalence of loss of vitality of different cements between porcelain-veneered zirconia and monolithic zirconia prostheses

^b^ Pearson’s chi-squared test

^c^ Fishers’ exact test

^d^ No events in both groups

Table 5 shows the prevalence of minor or major chipping among tooth-supported PVZ or MZ prostheses, for when air abrasion prior to cementation was conducted or not. No difference was observed in the prevalence of chipping, either minor or major, when either PVZ or MZ were air abraded or not.

**Table 5:** Prevalence of minor and major chipping among tooth-supported porcelain-veneered zirconia or monolithic zirconia prostheses, for when pre-cementation air abrasion was conducted or not. Here are considered the cases with available information for both the use of pre-cementation air abrasion and the occurrence of chipping. The unit is the prosthesis

Air abrasion	Veneered zirconia			Monolithic zirconia		
	Minor chipping	Major chipping	Total number prostheses	Minor chipping	Major chipping	Total number prostheses
	*n* (%)	*n* (%)	*n*	*n* (%)	*n* (%)	*n*
No	231 (6.9)	80 (2.4)	3,354	6 (0.4)	1 (0.1)	1,466
Yes	72 (7.9)	30 (3.3)	910	1 (0.2)	1 (0.2)	640
***p* value**	0.286 ^a^	0.124 ^a^		0.683 ^b^	0.516 ^b^	

^a^ Pearson’s chi-squared test

^b^ Fishers’ exact test

A total of 216 prostheses were considered failure, and the mean time from prosthesis cementation to failure was not statistically significant different between the groups (*p* = 0.935; Mann–Whitney test). There was no clear concentration of failures in any period of the follow-up, either for PVZ or MZ prostheses (Tables 6 and 7, respectively). The 5-year CSR was lower for PVZ than for MZ prostheses. The survival was statistically significant different between groups (*p* = 0.007; log-rank test). As the number of cases entering the longest follow-up intervals was very low for both groups, the survival between the groups was compared after the follow-up was limited to 5 years, which still resulted in a statistically significant difference between the groups (*p* < 0.001; log-rank test). When the data were limited to follow-up up to 5 years, the CSR was 88.7% and 93.3% for PVZ and for MZ prostheses, respectively.

**Table 6:** Life-table survival analysis showing the cumulative survival rate of tooth-supported veneered zirconia prosthesis

Interval start time (years)	Number entering interval	Number withdrawing during interval	Number exposed to risk	Prosthesis failures	ISR (%)	CSR (%)	SE
0	4264	1	4263.5	10	99.8	99.8	0.1
1	4253	912	3797.0	21	99.4	99.2	0.1
2	3320	706	2967.0	32	98.9	98.1	0.2
3	2582	861	2151.5	53	97.5	95.7	0.4
4	1668	516	1410.0	14	99.0	94.8	0.5
5	1138	502	887.0	11	98.8	93.6	0.6
6	625	150	550.0	5	99.1	92.7	0.7
7	470	252	344.0	24	93.0	86.3	1.4
8	194	0	194.0	6	96.9	83.6	1.7
9	188	92	142.0	0	100.0	83.6	1.7
10	96	72	60.0	0	100.0	83.6	1.7
11	24	1	23.5	1	95.7	80.1	3.9
12	22	22	11.0	0	100.0	80.1	3.9

*ISR*, interval survival rate (survival rate within each interval); *CSR*, cumulative survival rate (cumulative proportion surviving at end of interval); *SE*, standard error

**Table 7:** Life-table survival analysis showing the cumulative survival rate of tooth-supported monolithic zirconia prosthesis

Interval start time (years)	Number entering interval	Number withdrawing during interval	Number exposed to risk	Prosthesis failures	ISR (%)	CSR (%)	SE
0	2106	0	2106.0	3	99.9	99.9	0.1
1	2103	697	1754.5	10	99.4	99.3	0.2
2	1396	277	1257.5	3	99.8	99.1	0.2
3	1116	370	931.0	8	99.1	98.2	0.4
4	738	405	535.5	7	98.7	96.9	0.6
5	326	177	237.5	3	98.7	95.7	0.9
6	146	132	80.0	0	100.0	95.7	0.9
7	14	9	9.5	3	68.4	65.5	14.4
8	2	0	2.0	2	0.0	0.0	0.0

*ISR*, interval survival rate (survival rate within each interval); *CSR*, cumulative survival rate (cumulative proportion surviving at end of interval); *SE*, standard error

The estimated 5-year occurrence of minor chipping, major chipping, and catastrophic fracture for MZ prostheses were 0.076% (95% CI 0.042, 0.111, SE 0.018), 0.080% (95% CI 0.042, 0.118, SE 0.019), and 0.301% (95% CI 0.244, 0.357, SE 0.029), respectively (Table S1, see Supplementary material). The estimated 5-year occurrence of minor chipping, major chipping, and catastrophic fracture for PVZ prostheses were 10.445% (95% CI 10.253, 10.637, SE 0.098), 1.874% (95% CI 1.803, 1.945, SE 0.036), and 0.383% (95% CI 0.349, 0.417, SE 0.017), respectively (Table S2, see Supplementary material). The difference in the 5-year occurrence of minor and major chipping between MZ and PVZ prostheses was statistically significant (*p* < 0.001 and *p* = 0.002, respectively), but nor for the occurrence of catastrophic fracture (*p* = 0.122).

### Quality assessment

All included studies were classified as “good” (Table S3, see Supplementary material). In most cases the main issues in the publications were related to statistical methods not being well described and to the inclusion of non-consecutive patients in the studies.

## Discussion

The aim of the present review was to evaluate and compare the clinical outcomes between full-coverage tooth-supported MZ and PVZ FDPs. The results showed that MZ prostheses present higher survival rates than PVZ prostheses (*p* = 0.007). The reason for this difference can be due to the great difference in the occurrence of some complications that led to removal and/or replacement of the prosthesis. A much higher occurrence of porcelain chipping/fracture, framework fracture, and vitality loss were observed among PVZ than among MZ restorations.

The group of PVZ restorations had a significantly higher mean follow-up time than the group of MZ restorations, namely, a mean of 48.2 and 38.4 months, respectively. This could be one of the reasons contributing to a higher rate of technical complications among PVZ in relation to the MZ ones. It is expected that the longer the mean follow-up time, the higher the risk of presenting more complications. However, it does not explain all the possible reasons for a higher occurrence of technical complications among PVZ prostheses.

PVZ prostheses are more prone to chip-off fractures than MZ prostheses—the latter do not have a veneering ceramic and are expected to have less chipping and fracture complications [87]. The greater prevalence of chipping among PVZ restorations can due to the mismatch in the thermal expansion between the porcelain and the zirconia framework, which leads to the development of residual stresses, which in turn leads to initiation of cracking and veneer chipping. During the cooling process, thermal gradients can develop in crowns and FDPs and create another source of residual stress [88–91]. In comparison to restorations made of PVZ, monolithic designs have a higher load-bearing capacity [92]. It is also important to point out that resin-based cements may increase the strength of zirconia restorations to fracture, with glass ionomer cements being usually associated with lower fracture strength [93–95], which is in accordance with the results of the present review—the rates of minor or major chipping, for either PVZ or MZ, were higher for when the prostheses were cemented with glass ionomer in comparison to adhesive resin cements. Another important issue is the distinction of zirconia crowns with different yttria contents (3 and 5 mol%), as 3 mol% zirconia crowns fracture at almost twice the loads of the 5 mol% zirconia crowns [93]. In this context, the authors of an in vitro study suggested that it would be wise to avoid pre-treatment of 5Y crowns with air abrasion, as this reduced the strength of these crowns, the same not happening with 3Y-crowns [93], although the authors of another in vitro study, although also showing a difference in fracture load between 3 and 5Y crowns, suggested that crowns fabricated from 5Y-Z may be particle abraded if luted with resin cements [94]. Unfortunately, it was not possible to make an analysis of either failure or complications in relation to the different yttria contents of the restorations, as detailed information about the zirconia material used was not always provided. For example, there was information in some studies that either “Lava,” or “BruxZir,” or “Prettau Zirconia” was used, but with no further detail reported. This is an issue since dental zirconia from these three brands are available as either 3Y-TZP or 5Y-TZP.

The higher prevalence of framework fracture among PVZ than in MZ prostheses (0.8% vs. 0.2%, respectively) may be related to the inherent difference in the restoration design between these two types of zirconia restorations, as PVZ restorations present a thinner core of zirconia material than MZ restorations, which in turn influences the thickness of the crown/prosthesis margins [96]. Therefore, it may be expected that PVZ prosthesis would have a higher risk of framework fracture.

Teeth supporting PVZ prostheses presented a higher rate of vitality loss than teeth supporting MZ prostheses (1.5% vs. 0.3%, respectively), which could be related to the fact that veneer restorations are thicker, meaning that more underlying tooth structure needs to be removed [2], raising the risk of overheating the pulp, which is increased as the dentin thickness decreases. The mean tooth structure removal for full-crown restorations is greater for PVZ than for MZ restorations [97]. Dentin is an efficient insulator and will dissipate heat if an adequate amount of thickness remains. As such, trauma to the pulp tissue via heat is dependent on the proximity of the heat source to the pulp [98]. Greater extent of destruction of coronal and root structure was found to be a significant predictor of root canal therapy after single-crown placement [99]. It has been suggested that leaving 2 mm of dentinal thickness is sufficient to provide the pulp with protection from most restorative procedures [100]. Tooth vitality is suggested to contribute positively to the survival of single crowns and fixed dental prostheses [101].

The rate of decementation was higher when prostheses were cemented with zinc phosphate (5.0%)—only cases of PVZ prostheses were cemented with this type of cement. The zinc phosphate cement should not be recommended due to unfavorable properties such as brittleness and water solubility [102]. When it comes to the most commonly used cements, decementation was more commonly seen among prostheses cemented with adhesive resin than with glass ionomer in the MZ group. This goes against the results of an in vitro study, in which it was observed that adhesive resin cements present higher bond strength than glass ionomer cements in the bonding between zirconia and dentin [103]. However, a study observed no difference in the clinical performance for the cementation of zirconia copings between these two types of cement after 48 months [104]. As there is still no clear consensus about the difference in performance between the use of these two cements for zirconia restorations, one can only hypothesize that the possible cause for these findings could be related to the downsides of resin cements. Adhesive bonding has the disadvantage of being technique sensitive, as multiple steps of pretreatment are required and the prepared surface can easily get contaminated [93, 105]. Moreover, zirconia does not bond to resin-based cement as strongly as a silica-based ceramic [93, 106]. These factors could possibly have influenced the results.

No cases of decementation were observed among prostheses cemented with resin-modified glass ionomer, even when the inner surfaces of the prostheses were not air abraded previously to cementation. In general, the rate of decementation for zirconia restorations cemented with either conventional glass ionomer or adhesive resin cements was higher when the inner surface of the prostheses were air abraded before cementation. However, it seems that there is still some controversy whether air abrasion alters the shear bond strength of zirconia ceramic restorations [107]. Nevertheless, sandblast damage introduced into the ceramic undersurfaces causes further reductions in strength levels, with surface abrasion treatments possibly being an important degrading factor in long-term performance of all-ceramic crowns [108].

The problem with decementation when adhesive cements are used may be due to the already aforementioned reasons, although in vitro studies showed that resin-modified glass-ionomer cement presented the same level of retentive quality or the same mean removal stress as resin luting agents [109–111]. Moreover, similar clinical outcomes for the cementation of zirconia restorations between these cements were observed in a clinical study [112].. However, there is no clear consensus on the subject, as other in vitro studies showed that resin-modified glass ionomer cements present lower mean bond strengths between zirconia and dentin than resin-based luting cements [103, 113]. The results were not favorable for the conventional glass ionomer cement though. Compared to conventional glass ionomer, resin-modified glass ionomer cements have been showing better clinical performance [114].

The present review is not without limitations. First of all, most of the included studies were retrospective reports, which inherently results in flaws, manifested by the gaps in information and incomplete records. The number of technical complications observed is very probably underestimated, since not all studies reported data on all types of complications. Second, several professionals were involved in the treatment of these patients, which could have had some influence on the failure and complications rate. Third, much of the research in the field is limited by small cohort sizes and short follow-up periods, which could have led to an underestimation of the actual clinical outcomes.

## Conclusions

Tooth-supported PVZ FDPs present a lower survival than MZ FDPs (*p* = 0.007). The difference in the 5-year occurrence of minor (*p* < 0.001) and major chipping (*p* = 0.002) between MZ and PVZ prostheses was statistically significant, the same not happening with catastrophic fracture (*p* = 0.122). Loss of vitality happens more often with abutment teeth to PVZ prostheses. Decementation was not observed with resin-modified glass ionomer cement. Air abrasion does not seem to clinically decrease the risk of decementation.

### Supplementary Information

Below is the link to the electronic supplementary material.

Supplementary file1 (DOCX 70 KB)

## Data Availability

The authors confirm that the data supporting the findings of this study are available within the article [and/or] its supplementary materials.
