# Supplementary material for: Clinical outcomes of tooth-supported monolithic zirconia vs. porcelain-veneered zirconia fixed dental prosthesis, with an additional focus on the cement type: a systematic review and meta-analysis
Source: Clin Oral Investig. 2023 Aug 26;27(10):5755–69. doi: 10.1007/s00784-023-05219-4 (PMC10560185; doi:10.1007/s00784-023-05219-4)
Supplement: Supplementary file 1 — Supplementary file1 (DOCX 70 KB) [file 784_2023_5219_MOESM1_ESM.docx]

**SUPPLEMENTARY MATERIAL**

**a. References of the included studies**

**b. Estimated five-year occurrence of minor and major chipping, and catastrophic fracture, for tooth-supported monolithic and porcelain-veneered zirconia fixed dental prostheses**

**c. Quality assessment of the included studies**

a. References of the included studies

1. Barile G, Capodiferro S, Muci G, Carnevale A, Albanese G, Rapone B, Corsalini M. Clinical Outcomes of Monolithic Zirconia Crowns on Posterior Natural Abutments Performed by Final Year Dental Medicine Students: A Prospective Study with a 5-Year Follow-Up. Int J Environ Res Public Health. 2023 Feb 8;20(4):2943.
2. Beuer F, Edelhoff D, Gernet W, Sorensen JA. Three-year clinical prospective evaluation of zirconia-based posterior fixed dental prostheses (FDPs). Clin Oral Investig. 2009 Dec;13(4):445-51.
3. Beuer F, Stimmelmayr M, Gernet W, Edelhoff D, Güth JF, Naumann M. Prospective study of zirconia-based restorations: 3-year clinical results. Quintessence Int. 2010 Sep;41(8):631-637.
4. Burke FJ, Crisp RJ, Cowan AJ, Lamb J, Thompson O, Tulloch N. Five-year clinical evaluation of zirconia-based bridges in patients in UK general dental practices. J Dent. 2013 Nov;41(11):992-9.
5. Cehreli MC, Kökat AM, Akça K. CAD/CAM Zirconia vs. slip-cast glass-infiltrated Alumina/Zirconia all-ceramic crowns: 2-year results of a randomized controlled clinical trial. J Appl Oral Sci. 2009 Jan-Feb;17(1):49-55.
6. Chaar MS, Passia N, Kern M. Ten-year clinical outcome of three-unit posterior FDPs made from a glass-infiltrated zirconia reinforced alumina ceramic (In-Ceram Zirconia). J Dent. 2015 May;43(5):512-7.
7. Christensen RP, Ploeger BJ. A clinical comparison of zirconia, metal and alumina fixed-prosthesis frameworks veneered with layered or pressed ceramic: a three-year report. J Am Dent Assoc. 2010 Nov;141(11):1317-29.
8. Dhima M, Paulusova V, Carr AB, Rieck KL, Lohse C, Salinas TJ. Practice-based clinical evaluation of ceramic single crowns after at least five years. J Prosthet Dent. 2014 Feb;111(2):124-30.
9. Dogan S, Raigrodski AJ, Zhang H, Mancl LA. Prospective cohort clinical study assessing the 5-year survival and success of anterior maxillary zirconia-based crowns with customized zirconia copings. J Prosthet Dent. 2017 Feb;117(2):226-232.
10. Ferrari M, Sorrentino R, Cagidiaco C, Goracci C, Vichi A, Gherlone E, Zarone F. Short-term clinical performance of zirconia single crowns with different framework designs: 3-year clinical trial. Am J Dent. 2015 Aug;28(4):235-40.
11. Forrer FA, Schnider N, Brägger U, Yilmaz B, Hicklin SP. Clinical performance and patient satisfaction obtained with tooth-supported ceramic crowns and fixed partial dentures. J Prosthet Dent. 2020 Oct;124(4):446-453.
12. Gardell E, Larsson C, von Steyern PV. Translucent Zirconium Dioxide and Lithium Disilicate: A 3-Year Follow-up of a Prospective, Practice-Based Randomized Controlled Trial on Posterior Monolithic Crowns. Int J Prosthodont. 2021 Mar-Apr;34(2):163-172.
13. Gherlone E, Mandelli F, Capparè P, Pantaleo G, Traini T, Ferrini F. A 3 years retrospective study of survival for zirconia-based single crowns fabricated from intraoral digital impressions. J Dent. 2014 Sep;42(9):1151-5.
14. Grohmann P, Bindl A, Hämmerle C, Mehl A, Sailer I. Three-unit posterior zirconia-ceramic fixed dental prostheses (FDPs) veneered with layered and milled (CAD-on) veneering ceramics: 1-year follow-up of a randomized controlled clinical trial. Quintessence Int. 2015 Nov-Dec;46(10):871-80.
15. Groten M, Huttig F. The performance of zirconium dioxide crowns: a clinical follow-up. Int J Prosthodont. 2010 Sep-Oct;23(5):429-31.
16. Gseibat M, Sevilla P, Lopez-Suarez C, Rodríguez V, Peláez J, Suárez MJ. Prospective Clinical Evaluation of Posterior Third-Generation Monolithic Zirconia Crowns Fabricated with Complete Digital Workflow: Two-Year Follow-Up. Materials (Basel). 2022 Jan 17;15(2):672.
17. Habibi Y, Dawid MT, Waldecker M, Rammelsberg P, Bömicke W. Three-year clinical performance of monolithic and partially veneered zirconia ceramic fixed partial dentures. J Esthet Restor Dent. 2020 Jun;32(4):395-402.
18. Hammoudi W, Trulsson M, Svensson P, Smedberg JI. Long-term results of a randomized clinical trial of 2 types of ceramic crowns in participants with extensive tooth wear. J Prosthet Dent. 2022 Feb;127(2):248-257.
19. Hansen TL, Schriwer C, Øilo M, Gjengedal H. Monolithic zirconia crowns in the aesthetic zone in heavy grinders with severe tooth wear - An observational case-series. J Dent. 2018 May;72:14-20.
20. Heller H, Sreter D, Arieli A, Beitlitum I, Pilo R, Levartovsky S. Survival and Success Rates of Monolithic Zirconia Restorations Supported by Teeth and Implants in Bruxer versus Non-Bruxer Patients: A Retrospective Study. Materials (Basel). 2022 Jan 22;15(3):833.
21. Håff A, Löf H, Gunne J, Sjögren G. A retrospective evaluation of zirconia-fixed partial dentures in general practices: an up to 13-year study. Dent Mater. 2015 Feb;31(2):162-70.
22. Ioannidis A, Bindl A. Clinical prospective evaluation of zirconia-based three-unit posterior fixed dental prostheses: Up-to ten-year results. J Dent. 2016 Apr;47:80-5.
23. Kasem AT, Ellayeh M, Özcan M, Sakrana AA. Three-year clinical evaluation of zirconia and zirconia-reinforced lithium silicate crowns with minimally invasive vertical preparation technique. Clin Oral Investig. 2023 Apr;27(4):1577-1588.
24. Kitaoka A, Akatsuka R, Kato H, Yoda N, Sasaki K. Clinical Evaluation of Monolithic Zirconia Crowns: A Short-Term Pilot Report. Int J Prosthodont. 2018 Mar/Apr;31(2):124-126.
25. Koenig V, Vanheusden AJ, Le Goff SO, Mainjot AK. Clinical risk factors related to failures with zirconia-based restorations: an up to 9-year retrospective study. J Dent. 2013 Dec;41(12):1164-74.
26. Koenig V, Wulfman C, Bekaert S, Dupont N, Le Goff S, Eldafrawy M, Vanheusden A, Mainjot A. Clinical behavior of second-generation zirconia monolithic posterior restorations: Two-year results of a prospective study with Ex vivo analyses including patients with clinical signs of bruxism. J Dent. 2019 Dec;91:103229.
27. Kollar A, Huber S, Mericske E, Mericske-Stern R. Zirconia for teeth and implants: a case series. Int J Periodontics Restorative Dent. 2008 Oct;28(5):479-87.
28. Konstantinidis I, Trikka D, Gasparatos S, Mitsias ME. Clinical Outcomes of Monolithic Zirconia Crowns with CAD/CAM Technology. A 1-Year Follow-Up Prospective Clinical Study of 65 Patients. Int J Environ Res Public Health. 2018 Nov 12;15(11):2523.
29. Konstantinidis IK, Jacoby S, Rädel M, Böning K. Prospective evaluation of zirconia based tooth- and implant-supported fixed dental prostheses: 3-year results. J Dent. 2015 Jan;43(1):87-93.
30. Le M, Dirawi W, Papia E, Larsson C. Clinical outcome of three different types of posterior all-ceramic crowns. A 3-year follow-up of a multicenter, randomized, controlled clinical trial. Int J Prosthodont. 2022 Dec 6.
31. Lops D, Mosca D, Casentini P, Ghisolfi M, Romeo E. Prognosis of zirconia ceramic fixed partial dentures: a 7-year prospective study. Int J Prosthodont. 2012 Jan-Feb;25(1):21-3.
32. Mikeli A, Walter MH, Rau SA, Raedel M, Raedel M. Three-year clinical performance of posterior monolithic zirconia single crowns. J Prosthet Dent. 2022 Dec;128(6):1252-1257.
33. Miura S, Yamauchi S, Kasahara S, Katsuda Y, Fujisawa M, Egusa H. Clinical evaluation of monolithic zirconia crowns: a failure analysis of clinically obtained cases from a 3.5-year study. J Prosthodont Res. 2021 Jun 30;65(2):148-154.
34. Molin MK, Karlsson SL. Five-year clinical prospective evaluation of zirconia-based Denzir 3-unit FPDs. Int J Prosthodont. 2008 May-Jun;21(3):223-7.
35. Monaco C, Caldari M, Scotti R; AIOP Clinical Research Group. Clinical evaluation of 1,132 zirconia-based single crowns: a retrospective cohort study from the AIOP clinical research group. Int J Prosthodont. 2013 Sep-Oct;26(5):435-42.
36. Monaco C, Llukacej A, Baldissara P, Arena A, Scotti R. Zirconia-based versus metal-based single crowns veneered with overpressing ceramic for restoration of posterior endodontically treated teeth: 5-year results of a randomized controlled clinical study. J Dent. 2017 Oct;65:56-63.
37. Naenni N, Bindl A, Sax C, Hämmerle C, Sailer I. A randomized controlled clinical trial of 3-unit posterior zirconia-ceramic fixed dental prostheses (FDP) with layered or pressed veneering ceramics: 3-year results. J Dent. 2015 Nov;43(11):1365-70.
38. Ohlmann B, Eiffler C, Rammelsberg P. Clinical performance of all-ceramic cantilever fixed dental prostheses: results of a 2-year randomized pilot study. Quintessence Int. 2012 Sep;43(8):643-8.
39. Ortorp A, Kihl ML, Carlsson GE. A 5-year retrospective study of survival of zirconia single crowns fitted in a private clinical setting. J Dent. 2012 Jun;40(6):527-30.
40. Pathan MS, Kheur MG, Patankar AH, Kheur SM. Assessment of Antagonist Enamel Wear and Clinical Performance of Full-Contour Monolithic Zirconia Crowns: One-Year Results of a Prospective Study. J Prosthodont. 2019 Jan;28(1):e411-e416.
41. Peláez J, Cogolludo PG, Serrano B, Lozano JF, Suárez MJ. A prospective evaluation of zirconia posterior fixed dental prostheses: three-year clinical results. J Prosthet Dent. 2012 Jun;107(6):373-9.
42. Pihlaja J, Näpänkangas R, Raustia A. Early complications and short-term failures of zirconia single crowns and partial fixed dental prostheses. J Prosthet Dent. 2014 Oct;112(4):778-83.
43. Pihlaja J, Näpänkangas R, Raustia A. Outcome of zirconia partial fixed dental prostheses made by predoctoral dental students: A clinical retrospective study after 3 to 7 years of clinical service. J Prosthet Dent. 2016 Jul;116(1):40-6.
44. Poggio CE, Dosoli R, Ercoli C. A retrospective analysis of 102 zirconia single crowns with knife-edge margins. J Prosthet Dent. 2012 May;107(5):316-21.
45. Pontevedra P, Lopez-Suarez C, Rodriguez V, Pelaez J, Suarez MJ. Randomized clinical trial comparing monolithic and veneered zirconia three-unit posterior fixed partial dentures in a complete digital flow: three-year follow-up. Clin Oral Investig. 2022 Jun;26(6):4327-4335.
46. Raigrodski AJ, Yu A, Chiche GJ, Hochstedler JL, Mancl LA, Mohamed SE. Clinical efficacy of veneered zirconium dioxide-based posterior partial fixed dental prostheses: five-year results. J Prosthet Dent. 2012 Oct;108(4):214-22.
47. Rinke S, Gersdorff N, Lange K, Roediger M. Prospective evaluation of zirconia posterior fixed partial dentures: 7-year clinical results. Int J Prosthodont. 2013 Mar-Apr;26(2):164-71.
48. Rinke S, Schäfer S, Lange K, Gersdorff N, Roediger M. Practice-based clinical evaluation of metal-ceramic and zirconia molar crowns: 3-year results. J Oral Rehabil. 2013 Mar;40(3):228-37.
49. Sagirkaya E, Arikan S, Sadik B, Kara C, Karasoy D, Cehreli M. A randomized, prospective, open-ended clinical trial of zirconia fixed partial dentures on teeth and implants: interim results. Int J Prosthodont. 2012 May-Jun;25(3):221-31.
50. Sailer I, Balmer M, Hüsler J, Hämmerle CHF, Känel S, Thoma DS. 10-year randomized trial (RCT) of zirconia-ceramic and metal-ceramic fixed dental prostheses. J Dent. 2018 Sep;76:32-39.
51. Salido MP, Martinez-Rus F, del Rio F, Pradies G, Özcan M, Suarez MJ. Prospective clinical study of zirconia-based posterior four-unit fixed dental prostheses: four-year follow-up. Int J Prosthodont. 2012 Jul-Aug;25(4):403-9.
52. Sax C, Hämmerle CH, Sailer I. 10-year clinical outcomes of fixed dental prostheses with zirconia frameworks. Int J Comput Dent. 2011;14(3):183-202.
53. Schmitt J, Goellner M, Lohbauer U, Wichmann M, Reich S. Zirconia posterior fixed partial dentures: 5-year clinical results of a prospective clinical trial. Int J Prosthodont. 2012 Nov-Dec;25(6):585-9.
54. Schmitt J, Wichmann M, Holst S, Reich S. Restoring severely compromised anterior teeth with zirconia crowns and feather-edged margin preparations: a 3-year follow-up of a prospective clinical trial. Int J Prosthodont. 2010 Mar-Apr;23(2):107-9.
55. Schmitter M, Mussotter K, Rammelsberg P, Gabbert O, Ohlmann B. Clinical performance of long-span zirconia frameworks for fixed dental prostheses: 5-year results. J Oral Rehabil. 2012 Jul;39(7):552-7.
56. Seidel A, Belli R, Breidebach N, Wichmann M, Matta RE. The occlusal wear of ceramic fixed dental prostheses: 3-Year results in a randomized controlled clinical trial with split-mouth design. J Dent. 2020 Dec;103:103500.
57. Serra-Pastor B, Loi I, Fons-Font A, Solá-Ruíz MF, Agustín-Panadero R. Periodontal and prosthetic outcomes on teeth prepared with biologically oriented preparation technique: a 4-year follow-up prospective clinical study. J Prosthodont Res. 2019 Oct;63(4):415-420.
58. Seydler B, Schmitter M. Clinical performance of two different CAD/CAM-fabricated ceramic crowns: 2-Year results. J Prosthet Dent. 2015 Aug;114(2):212-6.
59. Shi JY, Li X, Ni J, Zhu ZY. Clinical Evaluation and Patient Satisfaction of Single Zirconia-Based and High-Noble Alloy Porcelain-Fused-to-Metal Crowns in the Esthetic Area: A Retrospective Cohort Study. J Prosthodont. 2016 Oct;25(7):526-530.
60. Solá-Ruiz MF, Baixauli-López M, Roig-Vanaclocha A, Amengual-Lorenzo J, Agustín-Panadero R. Prospective study of monolithic zirconia crowns: clinical behavior and survival rate at a 5-year follow-up. J Prosthodont Res. 2021 Aug 21;65(3):284-290.
61. Solá-Ruiz MF, Leon-Martine R, Labaig-Rueda C, Selva-Otalaorrouchi E, Agustín-Panadero R. Clinical outcomes of veneered zirconia anterior partial fixed dental prostheses: A 12-year prospective clinical trial. J Prosthet Dent. 2022:S0022-3913(20)30714-9.
62. Tanaka S, Takaba M, Ishiura Y, Kamimura E, Baba K. A 3-year follow-up of ceria-stabilized zirconia/alumina nanocomposite (Ce-TZP/A) frameworks for fixed dental prostheses. J Prosthodont Res. 2015 Jan;59(1):55-61.
63. Tang Z, Zhao X, Wang H, Liu B. Clinical evaluation of monolithic zirconia crowns for posterior teeth restorations. Medicine (Baltimore). 2019 Oct;98(40):e17385.
64. Tang Z, Zhao X, Wang H. Quantitative analysis on the wear of monolithic zirconia crowns on antagonist teeth. BMC Oral Health. 2021 Mar 4;21(1):94.
65. Tanner J, Niemi H, Ojala E, Tolvanen M, Närhi T, Hjerppe J. Zirconia single crowns and multiple-unit FDPs-An up to 8 -year retrospective clinical study. J Dent. 2018 Dec;79:96-101.
66. Tartaglia GM, Sidoti E, Sforza C. Seven-year prospective clinical study on zirconia-based single crowns and fixed dental prostheses. Clin Oral Investig. 2015 Jun;19(5):1137-45.
67. Teichmann M, Wienert AL, Rückbeil M, Weber V, Wolfart S, Edelhoff D. Ten-year survival and chipping rates and clinical quality grading of zirconia-based fixed dental prostheses. Clin Oral Investig. 2018 Nov;22(8):2905-2915.
68. Tinschert J, Schulze KA, Natt G, Latzke P, Heussen N, Spiekermann H. Clinical behavior of zirconia-based fixed partial dentures made of DC-Zirkon: 3-year results. Int J Prosthodont. 2008 May-Jun;21(3):217-22.
69. Tsumita M, Kokubo Y, Ohkubo C, Sakurai S, Fukushima S. Clinical evaluation of posterior all-ceramic FPDs (Cercon): a prospective clinical pilot study. J Prosthodont Res. 2010 Apr;54(2):102-5.
70. Valenti M, Valenti A, Schmitz JH, Cortellini D, Canale A. Survival analysis up to 7 years of 621 zirconia monolithic single crowns with feather-edge margins fabricated with a cast-free workflow starting from intraoral scans: A multicentric retrospective study. J Prosthet Dent. 2022 Jul 15:S0022-3913(22)00358-4.
71. Vigolo P, Mutinelli S. Evaluation of zirconium-oxide-based ceramic single-unit posterior fixed dental prostheses (FDPs) generated with two CAD/CAM systems compared to porcelain-fused-to-metal single-unit posterior FDPs: a 5-year clinical prospective study. J Prosthodont. 2012 Jun;21(4):265-9.
72. Vult von Steyern P, Carlson P, Nilner K. All-ceramic fixed partial dentures designed according to the DC-Zirkon technique. A 2-year clinical study. J Oral Rehabil. 2005 Mar;32(3):180-7.
73. Waldecker M, Behnisch R, Rammelsberg P, Bömicke W. Five-year clinical performance of monolithic and partially veneered zirconia single crowns-a prospective observational study. J Prosthodont Res. 2022 Apr 27;66(2):339-345.
74. Worni A, Katsoulis J, Kolgeci L, Worni M, Mericske-Stern R. Monolithic zirconia reconstructions supported by teeth and implants: 1- to 3-year results of a case series. Quintessence Int. 2017;48(6):459-467.

**b. Estimated five-year occurrence of minor and major chipping, and catastrophic fracture, for tooth-supported monolithic and porcelain-veneered zirconia fixed dental prostheses**

Table S1. Estimated five-year occurrence of minor and major chipping, and catastrophic fracture, for tooth-supported **monolithic zirconia** fixed dental prostheses

| Study | Total exposure time (years) | Estimated annual rate (per 100 prosthesis years) | | | Estimated occurrence after 5 years (in %) | | |
| --- | --- | --- | --- | --- | --- | --- | --- |
|  |  | Minor chipping | Major chipping | Catastrophic fracture | Minor chipping | Major chipping | Catastrophic fracture |
| Barile 2023 | 200.0 | 0 | 0.500 | 0 | 0 | 2.500 | 0 |
| Gardell 2021 | 99.9 | 0 | 0 | 0 | 0 | 0 | 0 |
| Gseibat 2022 | 60.0 | 0 | 0 | 0 | 0 | 0 | 0 |
| Habibi 2020 | 115.5 | 0 | 0 | 0 | 0 | 0 | 0 |
| Hammoudi 2022 | 1902.4 | 0.053 | 0 | 0 | 0.263 | 0 | 0 |
| Hansen 2018 | 140.3 | 2.851 | 0 | 0.713 | 14.257 | 0 | 3.564 |
| Heller 2022 | 1898.8 | 0.632 | 0 | 0 | 3.160 | 0 | 0 |
| Kasem 2023 | 60.0 | 0 | 0 | 0 | 0 | 0 | 0 |
| Kitaoka 2018 | 52.0 | 0 | 0 | 0 | 0 | 0 | 0 |
| Koenig 2019 | 26.0 | 0 | 3.846 | 3.846 | 0 | 19.231 | 19.231 |
| Konstantinidis 2018 | 65.0 | 0 | 0 | 0 | 0 | 0 | 0 |
| Le 2022 | 171.2 | 0 | 0 | 0 | 0 | 0 | 0 |
| Mikeli 2022 | 90.0 | 0 | 0 | 0 | 0 | 0 | 0 |
| Miura 2021 | 80.0 | 0 | 0 | 2.500 | 0 | 0 | 12.500 |
| Pathan 2019 | 60.0 | 0 | 0 | 0 | 0 | 0 | 0 |
| Solá-Ruiz 2021 | 250.0 | 0 | 0 | 0 | 0 | 0 | 0 |
| Tang 2019 | 89.7 | 0 | 0 | 0 | 0 | 0 | 0 |
| Tang 2021 | 43.0 | 0 | 0 | 0 | 0 | 0 | 0 |
| Valenti 2022 | 4351.6 | 0 | 0 | 0.023 | 0 | 0 | 0.115 |
| Waldecker 2022 | 939.6 | 0 | 0 | 0 | 0 | 0 | 0 |
| Worn 2017i | 140.6 | 0 | 0 | 0 | 0 | 0 | 0 |
| Global summary estimate  (95% CI) |  |  |  |  | 0.076 (0.042, 0.111)  SE 0.018  *p* < 0.001 | 0.080 (0.042, 0.118)  SE 0.019  *p* < 0.001 | 0.301 (0.244, 0.357)  SE 0.029  *p* < 0.001 |

SE – standard error

Table S2. Estimated five-year occurrence of minor and major chipping, and catastrophic fracture, for tooth-supported **porcelain-veneered zirconia** fixed dental prostheses

| Study | Total exposure time  (years) | Estimated annual rate  (per 100 prosthesis years) | | | Estimated occurrence after 5 years  (in %) | | |
| --- | --- | --- | --- | --- | --- | --- | --- |
|  |  | Minor chipping | Major chipping | Catastrophic fracture | Minor chipping | Major chipping | Catastrophic fracture |
| Beuer 2009 | 69.9 | 0 | 0 | 1.430 | 0 | 0 | 7.150 |
| Beuer 2010 | 198.6 | 2.518 | 0 | 0 | 12.591 | 0 | 0 |
| Burke 2013 | 170.6 | 4.103 | 0.586 | 0 | 20.515 | 2.931 | 0 |
| Cehreli 2009 | 60.0 | 0 | 0 | 0 | 0 | 0 | 0 |
| Chaar 2015 | 630.5 | 1.110 | 1.269 | 0.317 | 5.551 | 6.344 | 1.586 |
| Christensen 2010 | 489.0 | 14.519 | 5.317 | 0.409 | 72.597 | 26.585 | 2.045 |
| Dhima 2014 | 85.4 | 0 | 3.513 | 0 | 0 | 17.564 | 0 |
| Dogan 2017 | 97.8 | 0 | 0 | 0 | 0 | 0 | 0 |
| Ferrari 2015 | 270.0 | 1.481 | 0 | 0.741 | 7.407 | 0 | 3.704 |
| Forrer 2020 | 341.3 | 1.172 | 0 | 0 | 5.860 | 0 | 0 |
| Gherlone 2014 | 258.0 | 10.078 | 0 | 0 | 50.388 | 0 | 0 |
| Grohmann 2015 | 65.5 | 3.053 | 6.105 | 0 | 15.263 | 30.525 | 0 |
| Groten 2010 | 124.3 | 0 | 1.610 | 0 | 0 | 8.048 | 0 |
| Habibi 2020 | 105.4 | 2.846 | 0 | 0.949 | 14.231 | 0 | 4.744 |
| Håff 2015 | 316.8 | 0.631 | 0.316 | 0 | 3.157 | 1.578 | 0 |
| Ioannidis 2016 | 359.1 | 1.671 | 2.785 | 0 | 8.354 | 13.924 | 0 |
| Koenig | 204.1 | 2.449 | 2.449 | 0.980 | 12.246 | 12.246 | 4.899 |
| Kollar 2008 | 61.8 | 0 | 0 | 0 | 0 | 0 | 0 |
| Konstantinidis 2015 | 60.0 | 8.333 | 0 | 0 | 41.667 | 0 | 0 |
| Lops 2012 | 156.0 | 0.641 | 0 | 0.641 | 3.205 | 0 | 3.205 |
| Molin 2008 | 95.0 | 0 | 0 | 0 | 0 | 0 | 0 |
| Monaco 2013 | 2830.0 | 1.731 | 0.459 | 0.035 | 8.657 | 2.297 | 0.177 |
| Monaco 2017 | 493.2 | 0 | 0.608 | 0.203 | 0 | 3.041 | 1.014 |
| Naenni 2015 | 60.0 | 3.333 | 8.333 | 0 | 16.667 | 41.667 | 0 |
| Ohlmann 2012 | 20.0 | 10 | 0 | 0 | 50 | 0 | 0 |
| Ortorp 2012 | 1025.0 | 0.195 | 0.488 | 0 | 0.976 | 2.439 | 0 |
| Peláez 2012 | 60.0 | 3.333 | 0 | 0 | 16.667 | 0 | 0 |
| Pihlaja 2014 | 384.0 | 0 | 0.521 | 0 | 0 | 2.604 | 0 |
| Pihlaja 2016 | 588.0 | 2.211 | 0 | 0 | 11.054 | 0 | 0 |
| Poggio 2012 | 177.5 | 1.127 | 0 | 0 | 5.634 | 0 | 0 |
| Pontevedra 2022 | 199.8 | 2.002 | 0 | 0 | 10.010 | 0 | 0 |
| Raigrodski 2012 | 100.0 | 2.000 | 2.000 | 0 | 10 | 10 | 0 |
| Rinke 2013-1 | 158.1 | 1.265 | 0.633 | 0 | 6.326 | 3.163 | 0 |
| Rinke 2013-2 | 693.0 | 2.742 | 0.577 | 0.577 | 13.709 | 2.886 | 2.886 |
| Sagirkaya 2012 | 307.1 | 0 | 0.326 | 0.977 | 0 | 1.628 | 4.885 |
| Sailer 2018 | 400.0 | 3.750 | 1.250 | 0.250 | 18.750 | 6.250 | 1.250 |
| Salido 2012 | 68.0 | 0 | 0 | 4.412 | 0 | 0 | 22.059 |
| Sax 2011 | 278.2 | 5.392 | 0.359 | 1.078 | 26.959 | 1.797 | 5.392 |
| Schmitt 2010 | 58.5 | 1.709 | 0 | 0 | 8.547 | 0 | 0 |
| Schmitt 2012 | 150.0 | 2.667 | 2.000 | 0.667 | 13.333 | 10 | 3.333 |
| Schmitter 2012 | 150.0 | 4.667 | 0.667 | 1.333 | 23.333 | 3.333 | 6.667 |
| Seidel 2020 | 90.0 | 0 | 0 | 0 | 0 | 0 | 0 |
| Serra-Pasto 2019r | 404.0 | 0.248 | 0 | 0.248 | 1.238 | 0 | 1.238 |
| Seydler 2015 | 62.4 | 0 | 0 | 0 | 0 | 0 | 0 |
| Shi 2016 | 142.1 | 2.111 | 2.111 | 0 | 10.554 | 10.554 | 0 |
| Solá-Ruiz 2022 | 342.1 | 1.754 | 0.292 | 0 | 8.770 | 1.462 | 0 |
| Tanaka 2015 | 65.8 | 1.520 | 0 | 0 | 7.601 | 0 | 0 |
| Tanner 2018 | 200.0 | 3.000 | 0 | 0.500 | 15.000 | 0 | 2.500 |
| Tartaglia 2015 | 2121.0 | 0.141 | 0 | 0 | 0.707 | 0 | 0 |
| Teichmann 2018 | 270.0 | 2.222 | 0.370 | 0 | 11.111 | 1.852 | 0 |
| Tinschert 2008 | 181.5 | 2.203 | 0 | 0 | 11.017 | 0 | 0 |
| Tsumita 2010 | 49.1 | 6.105 | 0 | 0 | 30.525 | 0 | 0 |
| Vigolo 2012 | 195.0 | 2.051 | 1.026 | 0 | 10.256 | 5.128 | 0 |
| Vult von Steyern 2005 | 46.0 | 6.522 | 0 | 0 | 32.609 | 0 | 0 |
| Global summary estimate  (95% CI) |  |  |  |  | 10.445 (10.253, 10.637)  SE 0.098  *p* < 0.001 | 1.874 (1.803, 1.945)  SE 0.036  *p* < 0.001 | 0.383 (0.349, 0.417)  SE 0.017  *p* < 0.001 |

SE – standard error

**c. Quality assessment of the included studies**

| Study | Year | Was the study question or objective clearly stated? | Was the study population clearly and fully described, including a case definition? | Were the cases consecutive? | Were the subjects comparable? | Was the intervention clearly described? | Were the outcome measures clearly defined, valid, reliable, and implemented consistently across all study participants? | Was the length of follow-up adequate? ^a^ | Were the statistical methods well-described? | Were the results well-described? | Total (n/9) |
| --- | --- | --- | --- | --- | --- | --- | --- | --- | --- | --- | --- |
| Barile | 2023 | 1 | 1 | 1 | 1 | 1 | 1 | 1 | 0 | 1 | 8/9 |
| Beuer | 2009 | 1 | 1 | 0 | 1 | 1 | 1 | 1 | 1 | 0 | 8/9 |
| Beuer | 2010 | 1 | 1 | 0 | 1 | 0 | 1 | 1 | 1 | 1 | 7/9 |
| Burke | 2013 | 1 | 1 | 1 | 1 | 0 | 1 | 1 | 0 | 1 | 7/9 |
| Cehreli | 2009 | 1 | 1 | 0 | 1 | 1 | 1 | 1 | 0 | 1 | 7/9 |
| Chaar | 2015 | 1 | 1 | 1 | 1 | 0 | 1 | 1 | 1 | 0 | 7/9 |
| Christensen | 2010 | 1 | 1 | 1 | 1 | 1 | 1 | 1 | 1 | 1 | 9/9 |
| Dhima | 2014 | 1 | 1 | 1 | 1 | 1 | 1 | 1 | 1 | 1 | 9/9 |
| Dogan | 2017 | 1 | 1 | 0 | 1 | 1 | 1 | 1 | 1 | 1 | 8/9 |
| Ferrari | 2015 | 1 | 1 | 1 | 1 | 1 | 1 | 1 | 1 | 1 | 9/9 |
| Forrer | 2020 | 1 | 1 | 1 | 1 | 1 | 1 | 1 | 1 | 1 | 9/9 |
| Gardell | 2021 | 1 | 1 | 1 | 1 | 0 | 1 | 1 | 1 | 0 | 7/9 |
| Gherlone | 2014 | 1 | 1 | 0 | 1 | 1 | 1 | 1 | 1 | 1 | 8/9 |
| Grohmann | 2015 | 1 | 1 | 1 | 1 | 1 | 1 | 1 | 1 | 1 | 9/9 |
| Groten | 2010 | 1 | 1 | 1 | 1 | 0 | 1 | 1 | 0 | 1 | 7/9 |
| Gseibat | 2022 | 1 | 1 | 1 | 1 | 1 | 1 | 1 | 1 | 1 | 9/9 |
| Habibi | 2020 | 1 | 1 | 1 | 1 | 0 | 1 | 1 | 0 | 1 | 7/9 |
| Håff | 2015 | 1 | 1 | 0 | 1 | 1 | 1 | 1 | 0 | 1 | 7/9 |
| Hammoudi | 2022 | 1 | 1 | 0 | 1 | 1 | 1 | 1 | 1 | 1 | 8/9 |
| Hansen | 2018 | 1 | 1 | 0 | 1 | 1 | 1 | 1 | 0 | 1 | 7/9 |
| Heller | 2022 | 1 | 1 | 1 | 1 | 1 | 1 | 1 | 0 | 1 | 8/9 |
| Ioannidis | 2016 | 1 | 1 | 0 | 1 | 1 | 1 | 1 | 1 | 1 | 7/9 |
| Kasem | 2023 | 1 | 1 | 1 | 1 | 1 | 1 | 1 | 1 | 1 | 9/9 |
| Kitaoka | 2018 | 1 | 1 | 0 | 1 | 1 | 1 | 1 | 0 | 1 | 7/9 |
| Koenig | 2013 | 1 | 1 | 0 | 1 | 1 | 1 | 1 | 1 | 0 | 7/9 |
| Koenig | 2019 | 1 | 1 | 0 | 1 | 1 | 1 | 1 | 1 | 0 | 7/9 |
| Kollar | 2008 | 1 | 1 | 1 | 1 | 1 | 1 | 1 | 1 | 1 | 9/9 |
| Konstantinidis | 2015 | 1 | 0 | 1 | 1 | 1 | 1 | 1 | 0 | 1 | 7/9 |
| Konstantinidis | 2018 | 1 | 0 | 1 | 1 | 1 | 1 | 1 | 1 | 1 | 8/9 |
| Le | 2022 | 1 | 1 | 1 | 1 | 1 | 1 | 1 | 1 | 1 | 9/9 |
| Lops | 2012 | 1 | 1 | 0 | 1 | 1 | 1 | 1 | 1 | 1 | 8/9 |
| Mikeli | 2022 | 1 | 0 | 0 | 1 | 1 | 1 | 1 | 1 | 1 | 7/9 |
| Miura | 2021 | 1 | 1 | 0 | 1 | 1 | 1 | 1 | 1 | 0 | 7/9 |
| Molin | 2008 | 1 | 1 | 1 | 1 | 1 | 1 | 1 | 0 | 1 | 8/9 |
| Monaco | 2013 | 1 | 1 | 1 | 1 | 1 | 1 | 1 | 1 | 1 | 9/9 |
| Monaco | 2017 | 1 | 1 | 1 | 1 | 1 | 1 | 1 | 1 | 1 | 9/9 |
| Naenni | 2015 | 1 | 1 | 1 | 1 | 1 | 1 | 1 | 1 | 1 | 9/9 |
| Ohlmann | 2012 | 1 | 1 | 1 | 1 | 1 | 1 | 1 | 1 | 1 | 9/9 |
| Ortorp | 2012 | 1 | 1 | 1 | 1 | 1 | 1 | 1 | 1 | 1 | 9/9 |
| Pathan | 2019 | 1 | 1 | 1 | 1 | 0 | 1 | 1 | 0 | 1 | 7/9 |
| Peláez | 2012 | 1 | 1 | 0 | 1 | 1 | 1 | 1 | 0 | 1 | 7/9 |
| Pihlaja | 2014 | 1 | 1 | 0 | 1 | 1 | 1 | 1 | 1 | 1 | 8/9 |
| Pihlaja | 2016 | 1 | 1 | 0 | 1 | 0 | 1 | 1 | 1 | 1 | 7/9 |
| Poggio | 2012 | 1 | 1 | 1 | 1 | 0 | 1 | 1 | 0 | 1 | 7/9 |
| Pontevedra | 2022 | 1 | 1 | 0 | 1 | 1 | 1 | 1 | 0 | 1 | 7/9 |
| Raigrodski | 2012 | 1 | 1 | 1 | 1 | 0 | 1 | 1 | 1 | 0 | 7/9 |
| Rinke | 2013 | 1 | 1 | 1 | 1 | 1 | 1 | 1 | 1 | 1 | 9/9 |
| Rinke-2 | 2013 | 1 | 1 | 0 | 1 | 1 | 1 | 1 | 0 | 1 | 7/9 |
| Sagirkaya | 2012 | 1 | 1 | 1 | 1 | 1 | 1 | 1 | 0 | 1 | 8/9 |
| Sailer | 2018 | 1 | 1 | 0 | 1 | 1 | 1 | 1 | 1 | 1 | 8/9 |
| Salido | 2012 | 1 | 1 | 1 | 1 | 1 | 1 | 1 | 1 | 1 | 9/9 |
| Sax | 2011 | 1 | 1 | 0 | 1 | 1 | 1 | 1 | 0 | 1 | 7/9 |
| Schmitt | 2010 | 1 | 1 | 0 | 1 | 1 | 1 | 1 | 1 | 0 | 7/9 |
| Schmitt | 2012 | 1 | 1 | 0 | 1 | 1 | 1 | 1 | 1 | 0 | 7/9 |
| Schmitter | 2012 | 1 | 1 | 0 | 1 | 1 | 1 | 1 | 1 | 1 | 8/9 |
| Seidel | 2020 | 1 | 1 | 1 | 1 | 1 | 1 | 1 | 1 | 1 | 9/9 |
| Serra-Pastor | 2019 | 1 | 1 | 0 | 1 | 1 | 1 | 1 | 1 | 1 | 8/9 |
| Seydler | 2015 | 1 | 1 | 0 | 1 | 1 | 1 | 1 | 1 | 1 | 8/9 |
| Shi | 2016 | 1 | 1 | 0 | 1 | 1 | 1 | 1 | 1 | 1 | 8/9 |
| Solá-Ruiz | 2021 | 1 | 1 | 1 | 1 | 1 | 1 | 1 | 1 | 1 | 9/9 |
| Solá-Ruiz | 2022 | 1 | 1 | 0 | 1 | 1 | 1 | 1 | 1 | 1 | 8/9 |
| Tanaka | 2015 | 1 | 1 | 0 | 1 | 1 | 1 | 1 | 1 | 1 | 8/9 |
| Tang | 2019 | 1 | 1 | 1 | 1 | 1 | 1 | 1 | 1 | 0 | 8/9 |
| Tang | 2021 | 1 | 1 | 1 | 1 | 1 | 1 | 1 | 1 | 0 | 8/9 |
| Tartaglia | 2015 | 1 | 1 | 0 | 1 | 1 | 1 | 1 | 0 | 1 | 7/9 |
| Teichmann | 2018 | 1 | 1 | 0 | 1 | 1 | 1 | 1 | 1 | 1 | 8/9 |
| Tinschert | 2008 | 1 | 1 | 1 | 1 | 1 | 1 | 1 | 1 | 1 | 9/9 |
| Tsumita | 2010 | 1 | 1 | 0 | 1 | 1 | 1 | 1 | 1 | 1 | 8/9 |
| Valenti | 2022 | 1 | 1 | 1 | 1 | 1 | 1 | 1 | 0 | 1 | 8/9 |
| Vigolo | 2012 | 1 | 1 | 1 | 1 | 1 | 1 | 1 | 0 | 1 | 8/9 |
| Vult von Steyern | 2005 | 1 | 1 | 0 | 1 | 1 | 1 | 1 | 1 | 1 | 8/9 |
| Waldecker | 2022 | 1 | 1 | 1 | 1 | 1 | 1 | 1 | 1 | 1 | 9/9 |
| Worni | 2017 | 1 | 1 | 1 | 1 | 1 | 1 | 1 | 1 | 1 | 9/9 |

^a^ 6 months of follow-up was chosen to be of adequate length.
